# Supplementary material for: Transcriptome Profile Alteration with Cadmium Selenide/Zinc Sulfide Quantum Dots in Saccharomyces cerevisiae
Source: Biomolecules. 2019 Oct 25;9(11):653. doi: 10.3390/biom9110653 (PMC6920935; doi:10.3390/biom9110653)
Supplement: Supplementary file 1 [file biomolecules-09-00653-s001.pdf]

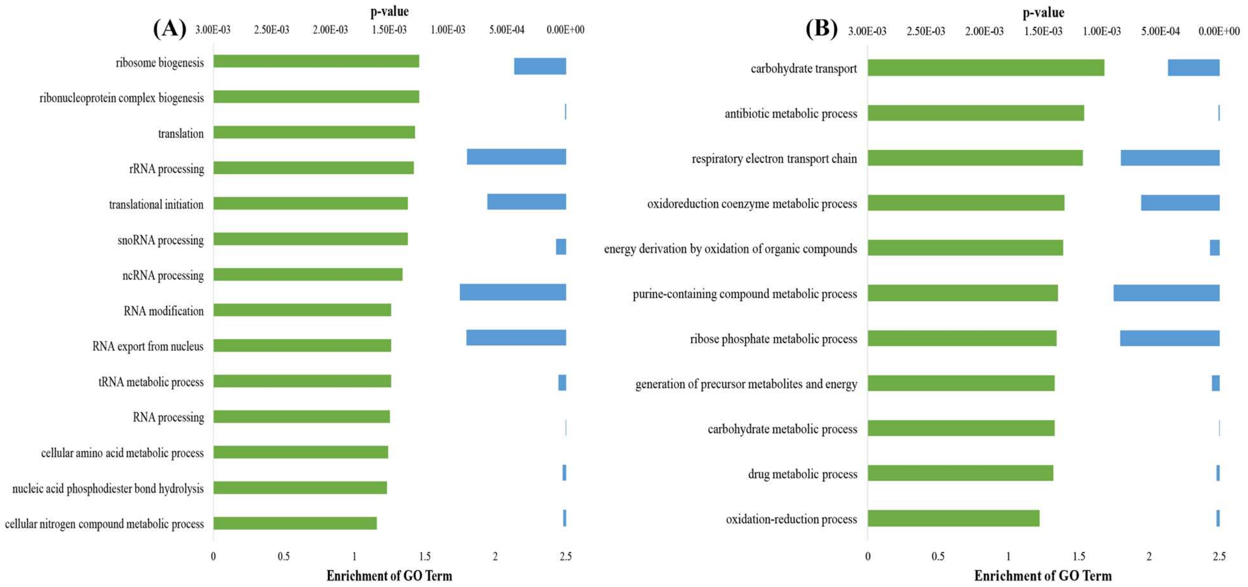

Figure S1. Enrichment and P-value of GO terms corresponding to each differentially expressed gene's individual biological process. Out of 4,478 genes with a q-value below 0.05, 2,839 genes with a fold change greater than or equal to 1.5 were incorporated. **(A)** The Enrichment and P-values from upregulated genes associated with their specific GO terms. **(B)** The Enrichment and P-values from downregulated genes associated with their specific GO terms.

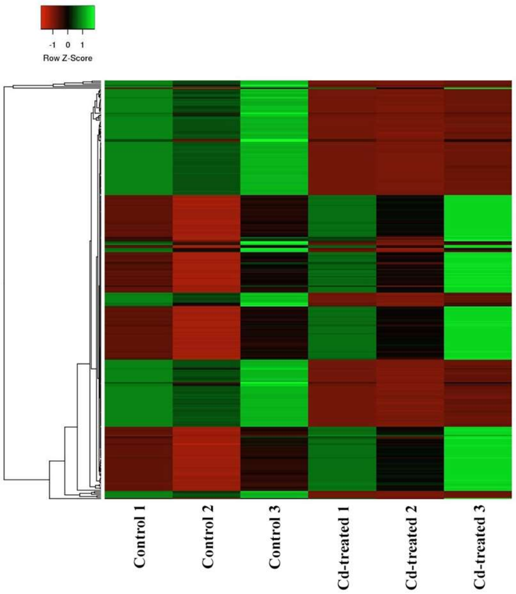

Figure S2. Heatmap of clustered gene expression data for 150 most up- and downregulated DEGs in response to CdSe/ZnS QD exposure. Rows represent genes hierarchically clustered with www.heatmapper.ca with the clustering method set to average linkage and distance measurement method set to Euclidean. Columns 1-3 represent normal gene expression data for non-treated samples and columns 4-6 represent gene expression data for CdSe/ZnS-treated (10 μg/mL) samples.

Table S1: GO terms corresponding to each differentially expressed gene's individual biological process. From a total of 4,478 statistically significant genes with a q-value of 0.05 or lower the top 150 genes with the greatest fold changes in expression are incorporated. The quantification of upregulated genes associated with their specific GO terms. From the 1,560 significantly upregulated genes the 150 most differentially expressed genes are incorporated in the chart.

GO term	# of genes	Corresponding genes
cellular component organization or biogenesis such as ribosomal subunit biogenesis and its assembly to form functional ribosomes	102	ECM16, NSA2, DBP8, FAF1, LCP5, RRB1, MAK16, KRR1, PUF6, NOP56, NOP7, UTP14, SPB1, RPA43, KRI1, UTP13, NOP4, RPA190, ALB1, RIX7, NOG1, NOP1, UTP8, RIX1, RLP7, NSA1, RPF2, YVH1, PRP43, RPA34, ENP2, NOP58, RRP36, HCA4, RRS1, DBP3, OGG1, RPA49, NSR1, NAN1, RRN11, UTP6, DRS1, YTM1, MRT4, RCL1, NOG2, ESF2, UTP5, UTP23, TSR1, SYO1, UTP25, MRD1, NOC3, PWP2, CGR1, IPI1, IPI3, RPF1, RRP8, RSA4, REI1, BUD22, NUG1, SSF1, DHR2, NOC2, BRX1, RNT1, UTP20, REX4, NOP13, ENP1, DIM1, SDA1, RRP5, NOP12, KRE33, RPC19, DBP2, FAL1, NOP15, NIP7, RPA135, LTV1, SAS10, UTP21, BUD27, NOP14, IMP4, HAS1, UTP4, ROK1, UTP11, MAK21, ERB1, RRP12, NOP2, SNU13, RPA12, MAK5
ribosomal RNA metabolic process/processing	95	DBP8, NSA2, CGR1, ECM16, FAF1, LCP5, IPI1, RRB1, IPI3, MAK16, KRR1, RPF1, RRP8, PWP1, NOP7, NOP56, UTP14, SPB1, NUG1, BUD22, RPA43, DHR2, SSF1, BRX1, KRI1, UTP13, RNT1, UTP20, REX4, RPA190, NOP4, ENP1, DUS1, DIM1, NOG1, TRM1, NOP1, UTP8, RIX1, RRP5, RLP7, NSA1, RPF2, NOP12, KRE33, PRP43, RPA34, ENP2, NOP58, RRP36, RPC19, TRM11, DBP2, FAL1, HCA4, NOP15, MMS1, RRS1, NIP7, DBP3, RPA135, NSR1, RPA49, BFR2, NAN1, MTR4, RRN11, UTP6, SAS10, UTP21, DRS1, YTM1, RCL1, MRT4, NOP14, ESF2, UTP5, IMP4, HAS1, UTP23, UTP4, ROK1, UTP11, TSR1, RRP12, ERB1, SNU13, NOP2, GCD10, UTP25, RPA12, MRD1, NOC3, MAK5, PWP2
maturation of SSU-rRNA	28	RRP36, ECM16, FAF1, LCP5, FAL1, NOP7, NAN1, BFR2, BUD22, DHR2, UTP13, UTP6, SAS10, NOP14, DIM1, UTP5, HAS1, UTP8, ROK1, UTP4, RRP5, TSR1, RRP12, SNU13, PRP43, UTP25, ENP2, PWP2
cleavage involved in rRNA processing	27	RRP36, DBP8, LCP5, RRS1, DBP3, KRR1, UTP14, MTR4, BRX1, KRI1, UTP13, UTP6, UTP20, SAS10, ENP1, NOP14, RCL1, ESF2, UTP23, ROK1, RRP5, RLP7, UTP11, TSR1, MRD1, PWP2, NOP58
ribosomal large subunit biogenesis	26	NSA2, NOP15, RRS1, NIP7, MAK16, PUF6, RRP8, NOP7, REI1, NOC2, NOP4, ALB1, YTM1, MRT4, RIX7, NOG1, HAS1, SDA1, RLP7, NSA1, SYO1, MAK21, ERB1, NOP2, PRP43, MAK5
maturation of SSU-rRNA from tricistronic rRNA transcript (SSU-rRNA, 5.8S rRNA, LSU-rRNA)	25	RRP36, ECM16, FAF1, LCP5, FAL1, NAN1, NOP7, BFR2, DHR2, UTP13, UTP6, SAS10, NOP14, DIM1, UTP5, HAS1, UTP8, UTP4, TSR1, RRP12, SNU13, PRP43, UTP25, ENP2, PWP2
nucleocytoplasmic transport	20	RIX7, NOG2, NMD3, SRP40, NOG1, RRS1, UTP8, SDA1, RPF1, RIX1, REI1, KAP123, YVH1, MTR4, SYO1, LTV1, NUG1, ARX1, ENP1, MRT4
maturation of LSU-rRNA	19	NSA2, IPI1, NIP7, MAK16, HAS1, RPF1, RLP7, NOP7, RPF2, NOP12, SPB1, ERB1, NUG1, SSF1, NOP2, SNU13, PRP43, NOP4, MAK5
rRNA 5'-end processing	18	DBP8, ESF2, UTP23, ROK1, RRP5, RLP7, UTP11, UTP14, BRX1, UTP13, UTP6, SAS10, UTP20, MRD1, PWP2, NOP58, NOP14, RCL1

nucleobase-containing compound transport (i.e. RNA)	18	RIX7, NOG2, NMD3, RRS1, NOG1, UTP8, RPF1, SDA1, FUI1, RIX1, YVH1, KAP123, MTR4, NUG1, LTV1, ARX1, ENP1, MRT4
ribosomal small subunit biogenesis	12	RRP36, BUD22, LTV1, KRE33, IMP4, UTP25, HAS1, KRR1, ENP2, UTP21, NOP13, NOP14
ribosomal large subunit assembly	12	RPF2, YVH1, MAK21, IPI1, SSF1, BRX1, IPI3, REX4, DRS1, RIX1, RSA4, MRT4
ribosomal large subunit export from nucleus	10	RIX7, YVH1, NOG2, NUG1, NMD3, ARX1, RRS1, SDA1, RPF1, MRT4
ncRNA transcription	9	RPC19, RPA43, RPA34, RRN11, RNT1, RPA135, RPA190, RPA12, RPA49
Macromolecule methylation	9	SPB1, TRM11, DIM1, NOP2, GCD10, TRM1, NOP1, RMT2, RRP8
maturation of 5.8S rRNA	8	RPF2, NSA2, SPB1, MTR4, PRP43, MAK16, RPF1, MAK5
transcription by RNA polymerase I (i.e. rRNA synthesis)	8	RPC19, RPA43, RPA34, RRN11, RPA190, RPA135, RPA12, RPA49
nucleolar large rRNA transcription by RNA polymerase I	7	RPA43, RPA34, RRN11, RPA190, RPA135, RPA12, RPA49
positive regulation of transcription by RNA polymerase I	5	DHR2, UTP5, UTP8, UTP4, NAN1
assembly of large subunit precursor of preribosome	4	RPF2, NOP2, NIP7, NOG1
pre-replicative complex assembly involved in cell cycle DNA replication	4	IPI1, IPI3, RIX1, NOC3

Table S2: GO terms corresponding to each differentially expressed gene's individual biological process. From a total of 4,478 statistically significant genes with a q-value of 0.05 or lower the top 150 genes with the greatest fold changes in expression are incorporated. The quantification of downregulated genes associated with their specific GO terms. From the 1,279 significantly downregulated genes, the 150 most differentially expressed genes are incorporated in the chart.

**GO term**	**# of genes**	**Corresponding genes**
oxidation-reduction process	31	DSF1, GLC3, RGI1, GPH1, ALD6, GIP2, TSA2, ZTA1, CYB2, YNR073C, SDH1, RGI2, GPX1, CYC7, PGM2, ALD4, GAC1, BDH2, UGP1, ALD3, GDB1, NDE2, YML131W, SHH4, CTT1, AIM17, GSY1, ISF1, GTO1, ECM4, GUT2
carbohydrate metabolic process	24	DSF1, YMR196W, GLC3, GPH1, PGM2, GAC1, NQM1, GDB1, UGP1, NDE2, MAL32, GAL7, YMR084W, GSY1, AMS1, GIP2, MAL12, CAT8, GUT2, YNR073C, HXK1, ATH1, SUC2, EMI2
generation of precursor metabolites and energy	18	RGI1, GLC3, GPH1, CYC7, PGM2, GAC1, GSM1, UGP1, GDB1, SHH4, NDE2, GSY1, ISF1, GIP2, HXK1, EMI2, SDH1, RGI2
cofactor metabolic process	15	MHT1, ALD4, ACH1, NQM1, ALD3, ALD6, NDE2, CTT1, GTO1, ECM4, TSA2, GUT2, HXK1, YKL151C, EMI2
energy derivation by oxidation of organic compounds	14	RGI1, GLC3, GPH1, PGM2, CYC7, GAC1, GDB1, UGP1, NDE2, GSY1, ISF1, GIP2, RGI2, SDH1
cellular response to chemical stimulus	14	NCE103, GPX1, NQM1, GAD1, CAT8, XBP1, TSA2, SIP18, HSP12, ZTA1, USV1, ATH1, SSA4, DDR2
carbohydrate catabolic process	12	GAL7, AMS1, GPH1, MAL12, PGM2, GUT2, HXK1, ATH1, GDB1, SUC2, EMI2, MAL32

energy reserve metabolic process	10	GSY1, GLC3, RGI1, GIP2, GPH1, PGM2, GAC1, GDB1, UGP1, RGI2
cellular polysaccharide metabolic process	10	YMR084W, GSY1, GLC3, GIP2, GPH1, PGM2, GAC1, UGP1, GDB1, SUC2
polysaccharide metabolic process	10	YMR084W, GSY1, GLC3, GIP2, GPH1, PGM2, GAC1, GDB1, UGP1, SUC2
response to oxidative stress	10	CTT1, NCE103, GPX1, GAD1, XBP1, HSP12, TSA2, ZTA1, NQM1, DDR2
antibiotic metabolic process	9	CTT1, TSA2, PDC6, ALD4, ACH1, ALD6, NDE2, SDH1, SHH4
glycogen metabolic process	8	GSY1, GLC3, GIP2, GPH1, PGM2, GAC1, GDB1, UGP1
nicotinamide nucleotide metabolic process	8	ALD4, GUT2, HXK1, NQM1, YKL151C, ALD6, EMI2, NDE2
pyridine nucleotide metabolic process	8	ALD4, GUT2, HXK1, NQM1, YKL151C, ALD6, EMI2, NDE2
alcohol metabolic process	8	DSF1, PDC6, ALD4, GUT2, YNR073C, YAT2, ALD6, NDE2
cellular carbohydrate catabolic process	7	GPH1, MAL12, GUT2, ATH1, GDB1, SUC2, MAL32
polysaccharide (glycogen) biosynthetic process	7	YMR084W, GSY1, GLC3, PGM2, GAC1, GDB1, UGP1
disaccharide metabolic process	6	MAL12, PGM2, ATH1, UGP1, SUC2, MAL32
response to drug	6	CTT1, NCE103, CIN5, ATH1, PDR15, DDR2
response to salt stress	5	CTT1, MRK1, CIN5, USV1, ALD6
cellular oxidant detoxification	5	CTT1, GPX1, GTO1, ECM4, TSA2
ethanol metabolic process	4	PDC6, ALD4, ALD6, NDE2
cellular polysaccharide catabolic process	3	AMS1, GPH1, GDB1
acetate metabolic process	3	ALD4, ACH1, ALD6
mannitol metabolic process	2	DSF1, YNR073C
